# The role of neutrophil/lymphocyte ratio (NLR) and platelet/lymphocyte ratio (PLR) in the early diagnosis of pulmonary mucoepidermoid carcinoma and their clinical significance

**DOI:** 10.5937/jomb0-57818

**Published:** 2026-01-06

**Authors:** Limin Yang, Cuicui Zhao, Chunhua Ling, Wei Lei

**Affiliations:** 1 The First Affiliated Hospital of Soochow University, Department of Pulmonary and Critical Care Medicine, Suzhou, 215006, China

**Keywords:** neutrophil/lymphocyte ratio, platelet/lymphocyte ratio, pulmonary mucoepidermoid carcinoma, diagnosis, inflammatory response, odnos neutrofila i limfocita, odnos trombocita i limfocita, plućni mukoepidermoidni karcinom, dijagnoza, inflamatorni odgovor

## Abstract

**Background:**

Pulmonary mucoepidermoid carcinoma (PMEC) is often misdiagnosed due to the lack of specificity of clinical symptoms. The ratio of neutrophil/lymphocyte ratio (NLR) and the ratio of platelet/lymphocyte ratio (PLR) are used in the diagnosis and prognostic assessment of a variety of diseases. This paper aims to verify the auxiliary diagnostic value of NLR and PLR in the peripheral blood of PMEC, and calculate several indices to confirm the reliability of the hypothesis.

**Methods:**

A total of 26 patients with PMEC were enrolled as the case group, and 156 healthy patients were selected as the control group in this study according to the inclusion criteria and exclusion criteria. All clinical data were collected, and all subjects took blood from their fasting veins. The correlation analysis of NLR, PLR and tumour indicators was consistent with the normal distribution using Pearson analysis. The receiver operating characteristic (ROC) curve was used to calculate the diagnostic value of NLR and PLR.

**Results:**

NLR and PLR levels were significantly increased in patients with PMEC compared with healthy controls. PLR was positively correlated with the patient's stage, and NLR was independent of the patient's stage in PMEC patients. NLR was positively correlated with the patient's tumour size, and PLR was independent of the patient's tumour size. ROC curve analysis showed that NLR and PLR could be used as diagnostic indicators to distinguish patients with PMEC from normal people.

**Conclusions:**

NLR and PLR tests are simple, non-invasive, inexpensive, and have high patient compliance. As potential markers for screening PMEC patients, NLR and PLR have auxiliary value for further exploration and research, and are worth promoting in the clinical setting.

## Abstract (sr)

**Uvod:**

Plućni mukoepidermoidni karcinom (PMEC) se često pogrešno dijagnostikuje zbog nedostatka specifičnih kliničkih simptoma. Odnos neutrofila i limfocita (NLR) i odnos trombocita i limfocita (PLR) se koriste u dijagnostici i proceni prognoze različitih bolesti. Cilj ovog rada je da se proveri pomoćna dijagnostička vrednost NLR i PLR u perifernoj krvi kod PMEC, kao i da se izračunaju različiti pokazatelji radi potvrde pouzdanosti hipoteze.

**Metode:**

U studiju je uključeno ukupno 26 pacijenata sa PMEC kao eksperimentalna grupa a 156 zdravih osoba je izabrano kao kontrolna grupa, prema kriterijumima za uključivanje i isključivanje. Prikupljeni su svi klinički podaci, a uzorci krvi su uzimani iz vena u stanju gladovanja. Korelaciona analiza između NLR, PLR i tumorskih pokazatelja sprovedena je Pirsonovom analizom, s obzirom na to da su podaci pratili normalnu distribuciju. Za procenu dijagnostičke vrednosti NLR i PLR korišćena je ROC kriva.

**Rezultati:**

Nivoi NLR i PLR su bili značajno povišeni kod pacijenata sa PMEC u poređenju sa zdravim osobama. PLR je bio pozitivno korelisan sa stadijumom bolesti, dok NLR nije pokazivao zavisnost od stadijuma bolesti kod PMEC pacijenata. NLR je pokazivao pozitivnu korelaciju sa veličinom tumora, dok PLR nije pokazivao zavisnost od veličine tumora. Analiza ROC krive pokazala je da se NLR i PLR mogu koristiti kao dijagnostički pokazatelji za razlikovanje PMEC pacijenata od zdravih osoba.

**Zaključak:**

Testovi za NLR i PLR su jednostavni, neinvazivni, jeftini i imaju visoku prihvatljivost među pacijentima. Kao potencijalni markeri za skrining pacijenata sa PMEC, NLR i PLR imaju pomoćnu vrednost za dalje istraživanje i vredni su promocije u kliničkoj praksi.

## Introduction

Mucoepidermoid carcinoma (MEC) is a malignant tumor composed of different proportions of epidermoid cells, mucus cells, and intermediate cells, which tend to occur in the salivary glands [1]. Pulmonary cancer is the fastest growing malignant tumour in China in the past 30 years, and the current incidence and mortality rate rank first in malignant tumours [2]. Pulmonary MEC (PMEC) is relatively rare clinically, and it has been reported in the literature that accounts for 0.1-0.2% of all pulmonary cancers [3]. Due to its low incidence, most of the clinical studies are individual cases or small samples, and the clinical features, immunohistochemistry and other conditions are dim. Pathological diagnosis is the gold standard of disease diagnosis, and the general appearance of lung MEC is mostly round or irregular. The tumour is generally 3-5 cm in size, with a greyish white section and brittle materials. According to morphology and cell score, PMEC can be divided into two types, low level and high level [4].

The origin of PMEC is controversial, but it is generally believed that it originates from the ductal epithelium of the bronchial submucosal glands [5]. The histological findings were consistent with those of MEC originating from salivary glands, which consisted mainly of mucoid cells, epidermal cells and intermediate cells [6]. Because the tumour grows slowly, patients may remain asymptomatic for a long time, especially in the elderly [7]. Any specific clinical symptoms do not accompany PMEC, and the most common symptom is a cough. Other symptoms include bloody or whitish sputum, fever, hemoptysis, chest tightness, chest pain, hoarseness and dyspnea, but some patients have no obvious symptoms and are only diagnosed during physical examination [8]. Recent studies have found that age, lymph node enlargement and distant metastasis are statistically significant in the low-grade group and high-grade group, suggesting that MSCT and PET/CT images of PMEC patients show some characteristic manifestations [9] [10].

In recent years, numerous studies have reported that inflammation is closely related to the development and prognosis of tumours [11] [12]. The ratio of neutrophil/lymphocyte ratio (NLR) and the ratio of platelet/lymphocyte ratio (PLR) in peripheral blood, as markers reflecting the systemic inflammation of the body, are easily obtained in the clinic [13]. The progress of the tumour depends on the interaction between the host microenvironment and tumour cells, as well as inflammatory reaction, fibroblasts and vascular cells [14]. Inflammation contributes to cancer cell survival, multiplication and angiogenesis, can prevent adaptive immune responses and can alter the body's response to systemic therapy [15]. Chronic inflammation is closely associated with lung cancer development, progression and prognosis. Normal cells in the lung are repeatedly damaged and eventually become cancerous by inflammation, which may reduce the ability to remove carcinogens from the lung [16]. Clinical characteristics have limited ability to predict patient survival and response to treatment. NLR and PLR, which reflect the inflammatory and immune status of cancer patients, are novel indicators that have received increasing attention in recent years and are used in the diagnosis and prognostic assessment of a variety of diseases [17]. However, the value of clinical application is controversial, and there is no consistent standard for judgment.

Thus, this paper aims to verify the diagnostic value of NLR and PLR in the peripheral blood of PMEC, and calculate several indices to confirm the reliability of the hypothesis.

## Materials and methods

### Basic information

The First Affiliated Hospital of Soochow University admitted 26 patients with pathologically confirmed or immunohistochemical assistance confirmed as PMEC from January 2003 to January 2020. The Ethics Committee of the First Affiliated Hospital of Soochow University approved all patients to fill in the informed consent form and the study.

### Criteria and definitions

The following patients were included in this study: (1) patients who met the relevant diagnostic criteria in the guidelines, the primary lesion was confirmed by biopsy pathology; (2) patients with completely medical records; (3) patients who signed informed consent.

Exclusion criteria: (1) patients with other malignant tumours; (2) patients with severe endocrine diseases; (3) patients with mental system diseases and infectious diseases; (4) patients with heart, liver, kidney, brain and other important organ diseases; (5) patients with active inflammation.

### Data collection

All clinical data were collected, including age, sex, tumour site and size, lymph node stage, distant metastasis and TNM stage. All subjects were fasting in the morning, and 5 mL of venous blood was collected by vacuum blood collection tube (containing EDTA anticoagulant). Immediately after blood collection, mix it upside down for 5-8 times to prevent coagulation. All blood samples were centrifuged at 3000 rpm for 10 minutes within 2 hours after collection to separate plasma and blood cells. The plasma was divided into small portions and stored in the refrigerator at -80°C for subsequent detection. An automatic blood cell analyser tested blood cells (Nanjing Baden Medical Technology Co., Ltd.), and the results included neutrophil count, lymphocyte count, and platelet count. All blood samples were visually examined before testing to exclude hemolysis and hyperlipidemia.

### Statistical analysis

The measurement data for this study were expressed as averages, and the counting data were expressed in the form of rates or ratios. The correlation analysis of NLR, PLR and tumour indicators was consistent with the normal distribution using Pearson analysis. The receiver operating characteristic (ROC) curve was used to calculate the auxiliary diagnostic value of NLR and PLR. Statistical analysis was performed using SPSS 22.0 software. A two-sided t-test was used for two groups. Statistical significance was observed at P<0.05.

## Results

### Baseline information of patients

The basic information about the patients is shown in Table 1.

**Table 1:** Basic information of patients.

Number	Gender	Age	Tumor site	Tumor size<br>(cm)	Lymph node<br>staging	Distant<br>metastasis	TNM<br>staging
1	female	41	Left upper lung	4.9*3.6	N0	M0	IIB
2	male	36	Left main bronchus	2.4*1.9	N0	M0	IA3
3	female	35	Left lower lung	0.69*0.5	N0	M0	IA1
4	male	68	Left lung	3.1*2.1	N1	M0	IIB
5	female	34	Left upper lung	2.7*3.0	N0	M0	IIA
6	female	68	Right anterior mediastinum	4.3*2.2*3.5	N0	M0	IIA
7	male	46	Right upper lung	3.3*2.8	N0	M0	IB
8	male	58	Left upper lung	3.93*3.64	N0	M0	IB
9	male	75	Left upper lung	2.4*2.1	N0	M1a	IVA
10	female	39	Left lower lung	3.1*2.2	N0	M0	IB
11	male	61	Right lower lung	6.5*4.1	N0	M0	IIB
12	female	62	Right upper lung	6.4*5.5	N2	M1a	IVA
13	male	45	Left upper lung	5.0*4.0	N2	M0	IIIA
14	male	20	Upper end of carina	1.3*0.8	Nx	M0	IA1
15	male	53	Left upper lung	1.7	N0	M1a	IVA
16	male	65	Right lower lung	1.9	N0	M0	IVA
17	female	66	Left upper lung	2.1*1.6	N0	M0	IA1
18	male	76	Right lung	4.2*3.0	N2	M1a	IVA
19	female	50	Middle and lower lobe of right lung	3.0	N0	M0	IB
20	male	48	Longtushang	1.6	N0	M1c	IVB
21	male	69	Left upper lung	3.0	N2	M0	IIIA
22	male	28	Right middle and lower lung	3.2*2.5	N1	M0	IIB
23	male	49	Right bronchus	4.1*3.7	N2	M1a	IVA
24	male	71	Right lower lung	2.96*2.0	N1	M0	IIB
25	female	32	Left main bronchus	1.1*0.7	N0	M0	IIB
26	female	63	Left upper lung	4.1*2.7	N0	M1a	IVA

### Comparison of NLR and PLR levels between the PMEC group and the control group

**Figure 1:**
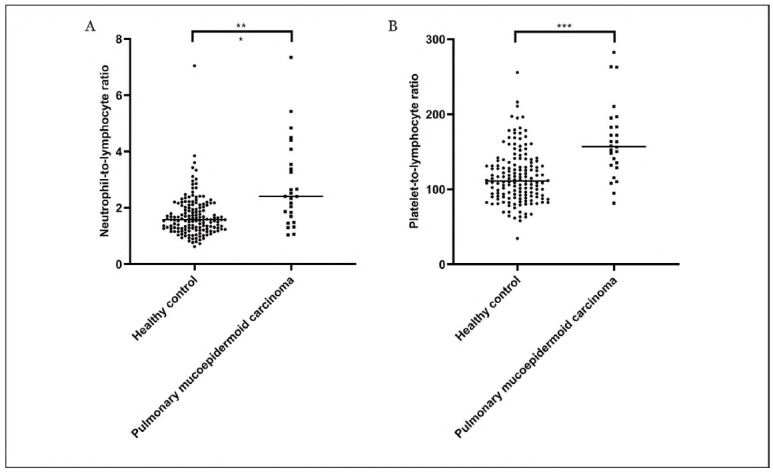
Comparison of NLR and PLR levels between PMEC group and the control group.

NLR and PLR were compared between the PMEC group and the control group. As shown in Figure 1, NLR and PLR levels were significantly increased in PMEC patients compared with healthy controls (***P<0.001).

### The relationship between NLR, PLR and tumour stage in PMEC patients

**Figure 2:**
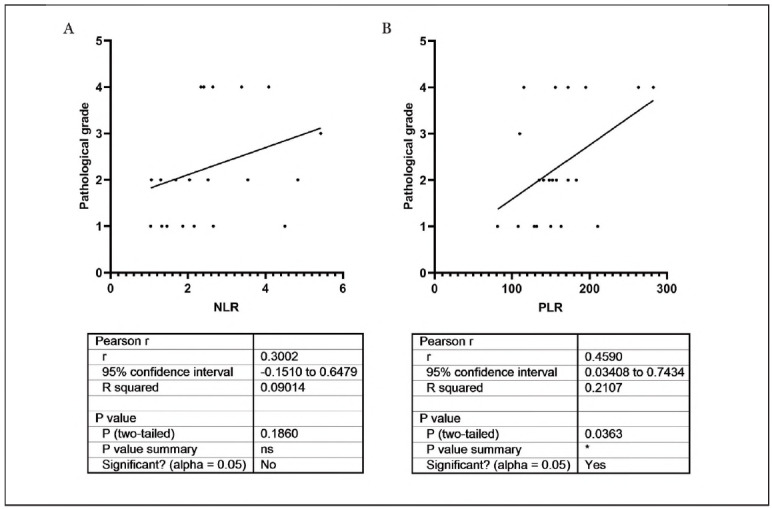
The relationship between NLR, PLR and tumour stage in PMEC patients.

To further explore the role of NLR and PLR in PMEC, the relationship between NLR, PLR and tumour stage in PMEC patients was tested. The results demonstrated that PLR was positively correlated with the patient's stage (95% confidence interval (CI): 0.3408-0.7434; r=0.4590, *P*<0.05), and NLR was independent of the patient's stage in PMEC patients (95% CI: -0.1510-0.6479; r=0.3002, *P*>0.05), (Figure 2).

### The relationship between NLR, PLR and tumour size in PMEC patients

**Figure 3:**
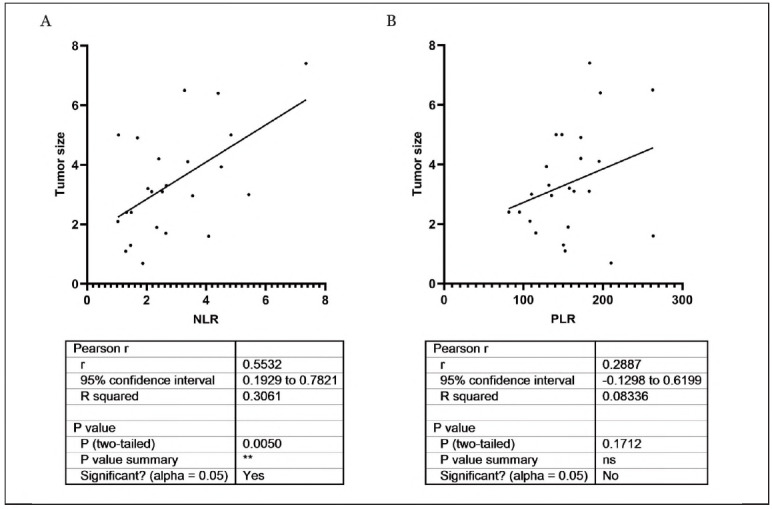
The relationship between NLR, PLR and tumour size in PMEC patients.

The relationship between NLR, PLR and tumour size in PMEC patients was also evaluated. As the results shown in Figure 3, NLR was positively correlated with the patient's tumor size (95% CI: 0.1929-0.7821; r=0.5532, *P*<0.05), and PLR was independent of the patient's tumor size (95% CI: -0.1298-0.6199; r=0.2887, *P*>0.05).

### ROC curve analysis of NLR and PLR

**Figure 4:**
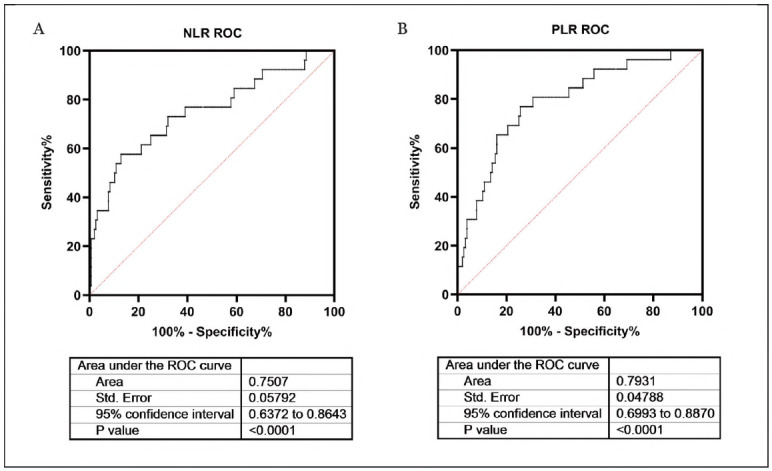
ROC curve analysis of NLR and PLR.

As shown in Figure 4, the ROC curve was used to calculate the diagnostic value of NLR and PLR. The AUC of NLR was 0.7507, 95% CI was 0.6372 to 0.8643, *P*<0.0001. The AUC of PLR was 0.7931, 95% CI was 0.6993 to 0.8870, *P*<0.0001. ROC curve analysis showed that NLR and PLR could be used as diagnostic indicators to distinguish patients with PMEC from normal people.

## Discussion

The present study found that NLR and PLR were significantly expressed in patients with PMEC. ROC results showed that NLR and PLR had higher AUC when used to distinguish patients with PMEC from normal people, which presented potential diagnostic value. PLR was positively associated with the patient's stage, and NLR was positively related to the patient's tumour size, suggesting that inflammatory lesions may be involved in the development of the PMEC.

NLR and PLR are two indicators related to systemic inflammation, and tumour occurrence, development, invasion and metastasis are related to inflammatory response [18] [19]. Although the role of NLR and PLR in the development of malignant tumours has not been clarified, tests for inflammatory substances are also commonly used clinically to aid diagnosis and reflect the condition. In the literature, neutropenia and lymphocytosis have been reported to coincide with greater stress and clinical improvement in the systemic inflammatory response. If the neutrophil count increases and the lymphocyte count decreases for more than one week, patients may develop serious complications such as multi-organ failure [20]. A host of neutrophils can promote tumour growth by secreting vascular endothelial growth factor and tumour cytokines, thereby assisting tumour invasion and metastasis [21]. A previous study has shown that neutrophils in non-small-cell lung cancer (NSCLC) suppress anti-tumour immune responses by suppressing the cytotoxic activity of immune cells [22]. Our findings showed that NLR levels were higher in the PMEC group than in the controls, and NLR was positively correlated with tumour size. The AUC was greater than 0.75, suggesting that PLR has potential diagnostic value in patients with PMEC.

PLR is one of the widely accepted clinical evaluation systems for evaluating inflammatory response and can better reflect the hypercoagulability and conduction of inflammatory pathways in patients [23]. A high platelet count may indicate an inflammatory response, such as the production of inflammatory mediators when some inflammatory factors stimulate macrophage proliferation [24]. PLR is an inflammatory marker that has been used as a prognostic factor for lung, liver and stomach cancer [25] [26]. Tumour cells induce platelet aggregation by releasing platelet agonists, which play a role in the progression of cancer [27]. As a dynamic reservoir of various factors, platelets secrete a large number of cytokines and transforming growth factors, which in turn stimulate tumour cell growth [28]. Tumours protect the tumour microenvironment by secreting platelet-retaining factors, thereby positively affecting their survival. Thrombocytosis and platelet activation are common in patients with malignant tumours, which leads to the release of pro-angiogenic proteins in alpha-granules that promote endothelial cell growth, survival, and proliferation, leading to angiogenesis and tumour cell survival [29]. PLR is a combination of these platelet and lymphocyte counts and may reflect a balance between tumour development and tumour suppression [30]. In this study, PMEC patients had a higher PLR compared with healthy individuals, and PLR was positively correlated with tumour stage, confirming the diagnostic value and role of PLR in the progression of PMEC.

To sum up, PMEC is a relatively rare type of lung cancer, and early diagnosis is very important to improve the prognosis of patients. This study found that NLR and PLR, as simple and accessible indicators, have potential value in the early screening and diagnosis of PMEC, especially for cases with atypical clinical symptoms. The correlation between PLR and tumour staging suggests that PLR can be used to evaluate the progress of the disease and assist clinicians in making more reasonable treatment plans. The correlation between NLR and tumour size suggests that it may reflect the invasion of the tumour and help to evaluate the prognosis. However, this study is limited by the small sample size, retrospective study design and single-centre data. Future research should focus on expanding the sample size for multicenter and prospective verification, exploring the joint application of NLR and PLR with other biomarkers to improve the accuracy of diagnosis and prognosis, studying their predictive value in the treatment of PMEC to guide individualised treatment, and deeply exploring their relationship with molecular typing of PMEC, so as to understand the occurrence and development mechanism of PMEC more comprehensively.

## Conclusion

PLR is positively correlated with tumour stage, and NLR is positively associated with tumour size. NLR and PLR have auxiliary diagnostic value in identifying patients with PMEC. In conclusion, NLR and PLR tests are simple, non-invasive, inexpensive, and have high patient compliance. As potential markers for screening PMEC patients, NLR and PLR have auxiliary diagnostic value for further exploration and research, and are worth promoting in the clinical setting.

## Dodatak

### Acknowledgements

Not applicable.

### Funding

This study was supported by the Gusu Youth Medical Talent (GSWS2020017, WL); the Science and Technology Program Project of Suzhou City(SL characI2022004); Bethune Charitable Foundation (BCF-XD-JC-20221205-05).

### Availability of data and materials

Not applicable.

### Authors' contributions

Limin Yang: Conceptualization; Writing - Original Draft; Writing - Review & Editing; Methodology.

Cuicui Zhao: Conceptualization; Writing - Original Draft; Writing - Review & Editing; Methodology.

Chunhua Ling: Project Administration; Funding Acquisition; Writing - Review & Editing.

Wei Lei: Project Administration; Funding Acquisition; Project Administration.

### Ethics approval and consent to participate

The Ethics Committee of The First Affiliated Hospital of Soochow University approved this study protocol. Written informed consent was provided prior to the study.

### Patient consent for publication

All the patients voluntarily participated in the study and provided informed consent before enrollment.

### Conflict of interest statement

All the authors declare that they have no conflict of interest in this work.
